# Supplementary material for: Splice-Junction-Based Mapping of Alternative Isoforms in the Human Proteome
Source: Cell Rep. Author manuscript; Available in PMC 2020 Jan 15. (PMC6961840; doi:10.1016/j.celrep.2019.11.026)
Supplement: 2 [file NIHMS1546469-supplement-2.zip › DF1/PXD010154/Testis/Testis_49_C1QBP_EQEVGQEDEAESDIFSIR.pdf]

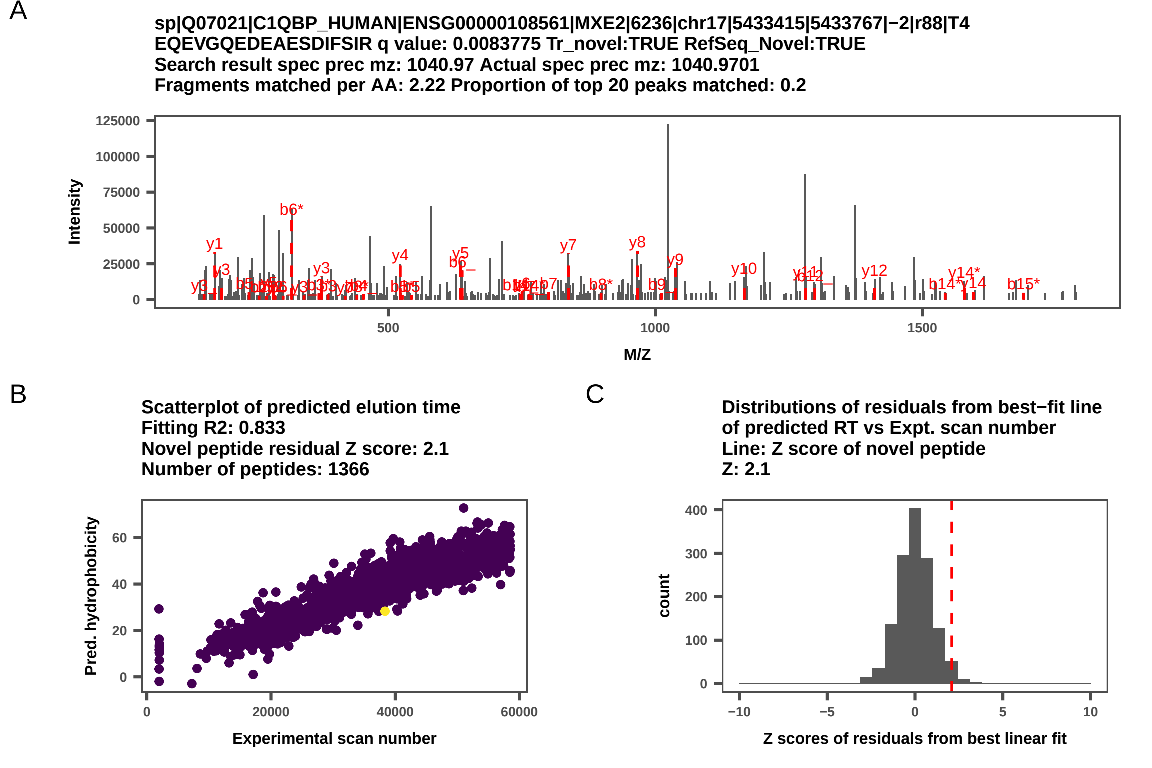
A
sp|Q07021|C1QBP_HUMAN|ENSG00000108561|MXE2|6236|chr17|5433415|5433767|−2|r88|T4
EQEVGQEDEAESDIFSIR q value: 0.0083775 Tr_novel:TRUE RefSeq_Novel:TRUE
Search result spec prec mz: 1040.97 Actual spec prec mz: 1040.9701
Fragments matched per AA: 2.22 Proportion of top 20 peaks matched: 0.2
Intensity
125000
100000
75000
50000
25000
0
b6*
y1
y3
y4
y5
y7
y8
y9
y10
y12
y14*
y14
b15*
500
1000
1500
M/Z
B
Scatterplot of predicted elution time
Fitting R2: 0.833
Novel peptide residual Z score: 2.1
Number of peptides: 1366
Pred. hydrophobicity
60
40
20
0
0
20000
40000
60000
Experimental scan number
C
Distributions of residuals from best−fit line
of predicted RT vs Expt. scan number
Line: Z score of novel peptide
Z: 2.1
count
400
300
200
100
0
−10
−5
0
5
10
Z scores of residuals from best linear fit
